# Patients’ knowledge of the indications for their medications – a scoping review

**DOI:** 10.1186/s12913-024-11685-7

**Published:** 2024-10-08

**Authors:** Cille Bülow, Stine Søndersted Clausen, Patrick Lundholm Thøgersen, Dagmar Abelone Dalin, Johanne Mølby Hansen, Karl Sebastian Johansson, Andreas Lundh, Mikkel Bring Christensen

**Affiliations:** 1 Department of Clinical Pharmacology, Copenhagen University Hospital - Bispebjerg and Frederiksberg, Copenhagen, Denmark; 2 The Research Unit for General Practice, Department of Public Health, University of Copenhagen, Copenhagen, Denmark; 3 Department of Respiratory Medicine and Infectious Diseases, Copenhagen University Hospital – Bispebjerg and Frederiksberg, Copenhagen, Denmark; 4 Cochrane Denmark & Centre for Evidence-Based Medicine Odense (CEBMO), Department of Clinical Research, University of Southern Denmark, Odense, Denmark; 5 Department of Clinical Medicine, Faculty of Health and Medical Sciences, University of Copenhagen, Copenhagen, Denmark

**Keywords:** Medication knowledge, Indications, Scoping review

## Abstract

**Background:**

Inadequate medication knowledge may contribute to inappropriate medication use and treatment harms. We aimed to map and synthesise the existing evidence on patients’ knowledge of the indications for their medications.

**Method:**

We searched MEDLINE, Embase, CINAHL, PsychInfo and the Cochrane Library for studies that assessed patients’ knowledge of the indications for their medications from inception to June 16, 2022. A pair of reviewers independently screened and extracted data on study characteristics, aims, and methods used to assess and report patients’ knowledge of the indications for their medications.

**Results:**

We included 99 studies conducted in 33 countries, published between 1979 and 2021, with 42,377 participants in total (median 126 participants [Interquartile range: 63–338]). Studies were observational (*n* = 77), experimental (*n* = 18), or qualitative interviews (*n* = 4). The exact question used to assess knowledge of the indications was reported in 27 studies and was phrased in 25 different ways. Knowledge of the indications was reported as a proportion of either 1) all participants (*n* = 65) or 2) the total number of medications used by all patients (*n* = 13). Sixteen studies used both reporting methods, while five only reported a proportion without specifying the denominator. Fourteen studies in various populations reported the number of participants with correct knowledge of all their medications, ranging from 19% (long-term psychiatric in-patients) to 87% (general practice patients).

**Conclusion:**

We did not identify any established scientific standard for assessing patients’ knowledge of the indications for their medications. The wide range of study methodologies and reporting styles observed call for a methodological consensus in this research field. Estimates of correct knowledge varied widely between studies, but whether this was due to differences in study populations or study methodology could not be determined. Furthermore, we did not identify any study investigating whether participants’ knowledge of the indications for their medications was associated with the quality, e.g. appropriateness, of their treatment.

**Supplementary Information:**

The online version contains supplementary material available at 10.1186/s12913-024-11685-7.

## Background

Medication literacy is defined as ‘the degree to which individuals can obtain, comprehend, communicate, calculate and process patient-specific information about their medications to make informed medication and health decisions in order to safely and effectively use their medications’ [1–3]. One component of medication literacy is an understanding of the reason for treatment i.e. the indication, which is defined by the European Medicines Agency as ‘a medical condition that a medicine is used for’ [4]. Previous studies have shown that poor knowledge about the indications for their medications are associated with patients having reduced adherence to therapy [5, 6].

Older individuals with polypharmacy and multimorbidity often face challenges in understanding medication indications, benefits, and harms, which can contribute to lower medication adherence [7, 8]. However, importantly, the same individuals with polypharmacy and multimorbidity are also often exposed to inappropriate medications, i.e. medications where the expected benefits for the individual patient do not outweigh the expected harms or costs [9]. Evaluating medication appropriateness requires ongoing communication between patients and healthcare providers, which can be particularly challenging for this population. Therefore, while medication literacy is crucial for engaging in shared decision-making and ensuring adherence [7], it may also be important for avoiding inappropriate prescriptions.

Several validated tools and methods exist to assess patients’ knowledge of their medications. These methods vary in complexity and focus, but they all aim to evaluate how well patients understand the different aspects of their medications, such as names, indications, dosage, timing, side effects, and storage. Some of the commonly used validated tools are: the Medicine Knowledge Assessment Form [10], MedTake [11], and the Medication Assessment Tool [12]. The tools are used in various healthcare settings to identify gaps in patients’ knowledge and provide targeted education to improve medication literacy and adherence, but their use depend on the context and the involved patient population. Although several studies have examined patients’ knowledge of the indications for their medications [5, 6, 13–28], the collected evidence on patients’ knowledge of their indications has not previously been systematically synthesised. In this scoping review, we aimed to map and synthesise the existing evidence on patients’ knowledge of the indications for their medications. Furthermore, we aimed to identify studies assessing the association between medication knowledge and the appropriateness of medication use.

## Methods

### Protocol and registration

The review protocol was developed using Joanna Briggs Institute’s (JBI) guidance for scoping reviews [29] and prospectively registered in Open Science Framework (https://osf.io/dv2hq/). The review is reported in line with the Preferred Reporting Items for Systematic Reviews and Meta-Analyses (PRISMA) extension for scoping reviews [30].

### Eligibility criteria

We included all quantitative and qualitative primary research studies, published in any language, that examined patients’ knowledge of the indications for their medications. Studies were excluded if they only examined patients’ knowledge of the indication for a part of their medications (e.g. only antihypertensives or medications for a selected indication). Conference abstracts without a subsequent full-text publication were excluded.

### Information sources

We searched MEDLINE (Ovid), Embase (Ovid), CINAHL (the Cumulative Index to Nursing and Allied Health Literature) (Ebsco), PsychInfo (Ebsco) and the Cochrane Library from inception to June 16, 2022.

### Search

We grouped search terms into four main themes; “patient”, “knowledge”, “medications”, and “indications”. These themes served as a structure for our search strategy, which was developed in collaboration with an information specialist (see Supplement 1). The search was developed in MEDLINE and translated for the other databases.

### Selection of studies

After removing duplicate records, a pair of review authors (CB, and KSJ or SSC) independently assessed all identified records for eligibility in two rounds using Covidence (www.covidence.org). Disagreements were resolved by discussion, and if consensus could not be reached, we involved an additional author (MC) as arbiter. First, we screened titles and abstracts for potential eligibility and then assessed the full-text manuscripts for final inclusion. If we identified conference abstracts without full-text publications, we contacted the first or last author to inquire about publication status (*n* = 10). We received answers from three authors, who all replied that their research had not been published in full text but only as conference abstracts.

### Data extraction

We extracted data independently in pairs (CB, and DAD, JMH or PLT) using a Microsoft Excel data sheet. When studies were published in languages other than English, the pair of review authors independently translated the study publications using Google Translate and extracted data independently. If there was any uncertainty about the translations, the text was translated to both English and Danish to minimise the risk of mistranslations. Disagreements were resolved by discussion, and if consensus could not be reached, we involved an additional author (MC) as the arbiter.

Data items.

- Study characteristics: publication year, journal name, study design (e.g. cross-sectional, experimental or qualitative), country of origin
- Study aim
- Participants: number, age, gender, setting, diseases, number of medications used, inclusion and exclusion criteria
- Methods: Description of the method used to assess patients’ knowledge of the indications for their medications
- Outcomes: Which outcomes were measured, how outcomes were measured
- Results: Patients’ knowledge of the indications for their medications

We anticipated including studies of various study designs (e.g. cross-sectional and experimental studies) which would require the use of multiple critical appraisal tools. This would make comparison between studies particularly challenging, and since the focus of our review was to map the evidence and not provide summary estimates, we decided not to undertake any critical study appraisal in line with guidance for conducting scoping reviews [29].

### Synthesis of results

Data synthesis was undertaken in three stages: (1) evidence mapping, (2) identification of evidence gaps and (3) a narrative synthesis of results. We charted data using frequencies and proportions. Specifically, we extracted data collection methods, how the studies assessed patients’ knowledge, if patients had access to help when their knowledge was assessed, and when knowledge was assessed as correct. We also analysed if the studies reported knowledge of indications at participant level or a medication level (i.e. out of the total number of medications used). Data were used to analyse patterns and identify knowledge gaps for methods used to access patients’ knowledge of the indications for their medications. We used Microsoft Excel to generate descriptive statistics.

## Results

### Study inclusion

**Fig. 1:**
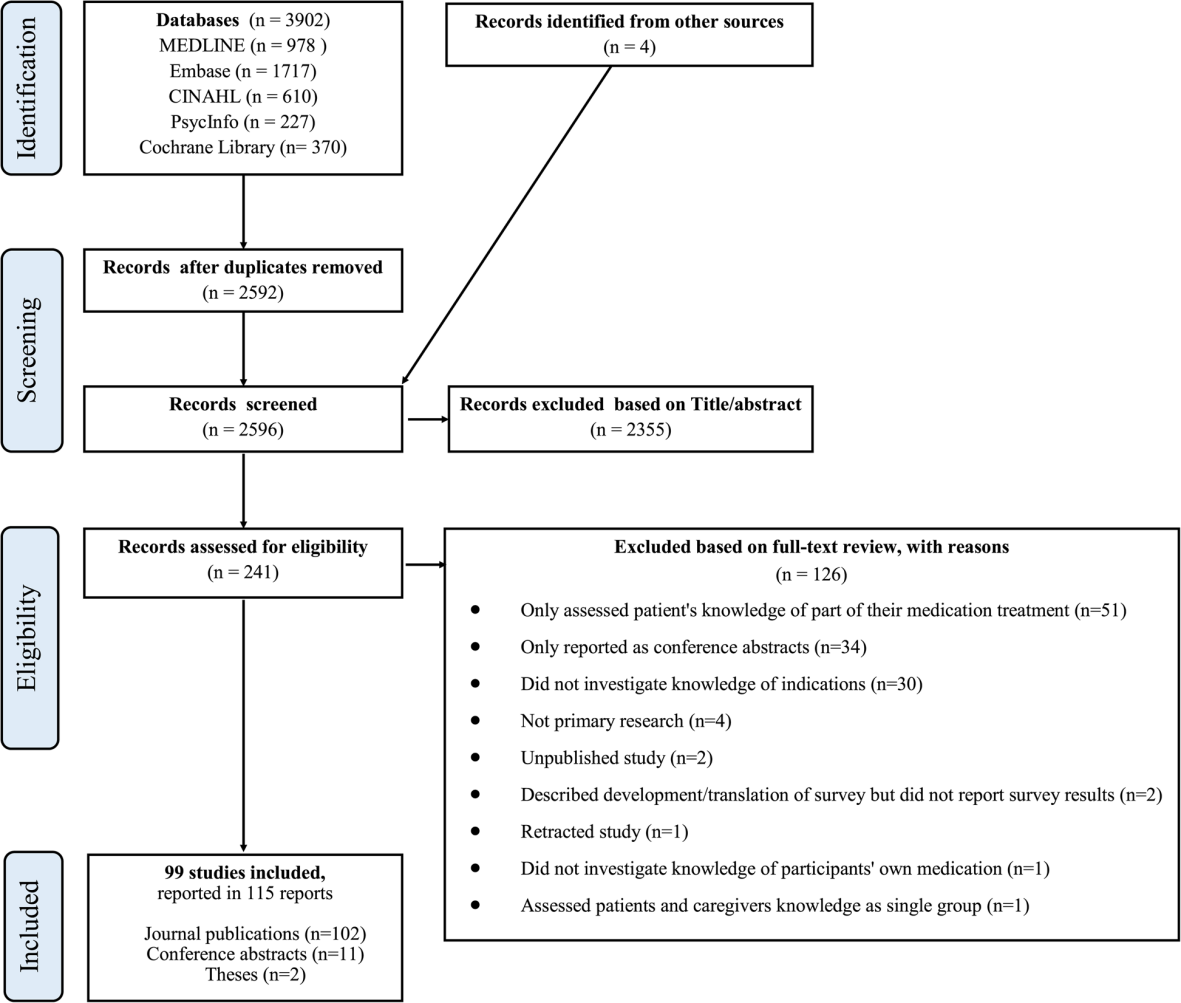
PRISMA flow diagram of the study selection

Our database search identified 2,592 unique records (Fig. 1). By screening titles and abstracts, we excluded 2,351 records and retained 241 as potentially relevant. We excluded 126 of these records based on full-text assessment and included 115, collectively describing 99 unique studies [5, 6, 15, 17, 20–22, 25, 27, 31–123]. Eleven studies were published both as conference abstracts and as full-text articles, and five studies were published in two separate journal publications (three published the same data in two different languages, and two studies had a slightly different focus in the two publications).

### Characteristics of included studies

Details of the 99 included studies are available in Supplement 2.

The studies were conducted in 33 countries and published between 1979 and 2021 in seven different languages, with 88 studies published in English (See Table 1). The studies included a total of 42,377 participants (median 126 participants, range 17 to 14,004 [Interquartile range (IQR): 63–338]). Age was an inclusion criterion in 51 studies, where 32 specifically included older adults (i.e. above 60 years). Number of medications used was a specific inclusion criterion in 33 studies (i.e. participants should take at least one medication (*n* = 19), two medications (*n* = 1), three medications (*n* = 5), four medications (*n* = 1), five medications (*n* = 6), or six medications (*n* = 1)). Sex was reported in 89 studies, and the proportion of females ranged between 3 and 95%. Morbidity was reported in 39 studies, most often as the proportion of participants with different diseases (*n* = 22) or Charlson comorbidity index score (*n* = 4). Of the 99 studies, 77 were observational (mainly cross-sectional (*n* = 74)), 18 were experimental, and 4 were qualitative interview studies.

**Table 1:** Characteristics of included studies

**Publications by:**	**Frequency** (*N* = 99)
**Region**	
Europe	39
Asia	19
Oceania	3
North America	34
South America	4
**Year published**	
Prior to 1990	7
1990–1999	13
2000–2009	20
2010–2019	47
2020–2021	12
**Language**	
English^a^	88
Other languages^a^	14
**Study design**	
*Observational*	*77*
Cross-sectional study	74
Cohort study	3
*Experimental*	*18*
Parallel group RCT	5
Cluster RCT	2
Before-and-after study	8
Non-randomised trial	3
*Qualitative*	*4*
Interview study	4
**Number of participants**	
Range	17 to 14,004
Median	126
IQR	63, 338
**Setting**	
Hospital	33
Patient’s home	20
Outpatient clinic	15
Primary practice	14
Multiple settings	5
Municipality	3
Not reported	3
Other	6
**Average age, years**	
≤ 49	7
50–59	10
60–69	24
70–79	30
≥ 80	9
Not reported	19
**Average number of medications**	
≤ 4	27
5–9	35
≥ 10	7
Not reported	30
**Female, %**	
≤ 39%	10
40–59%	46
≥ 60%	32
Not reported	11

*RCT* Randomised Clinical Trial, *IQR* Interquartile range

^a^Language: Dutch (*n* = 3), French (*n* = 2), German (*n* = 4), Japanese (*n* = 2), Portuguese (*n* = 2), Turkish (*n* = 1). Three studies published the same data in two different languages (English + Dutch (*n* = 2), English + Japanese (*n* = 1)

### Study aims

Table 2 shows the categories of aims identified in the studies. In 59 studies, the primary aim was to assess patient medication knowledge, 26 studies had medication knowledge as part of the aim, and 14 studies reported patients’ knowledge of indication for medications in the results, though this was not part of the study aim.

**Table 2:** Category of study aims

Aim	Frequency (*N* = 99)
To test the effect of an intervention	*18*
To identify factors affecting medication knowledge	*15*
To investigate medication knowledge in a specific population	*13*
To investigate factors associated with adherence	*9*
To assess medication knowledge in general	*9*
To investigate medication knowledge at a specific time point	*5*
To compare medication knowledge between different types of medications	*4*
To compare medication knowledge between different groups of patients	*3*
Other	*23*

### Methods used to assess knowledge of indications

**Fig. 2:**
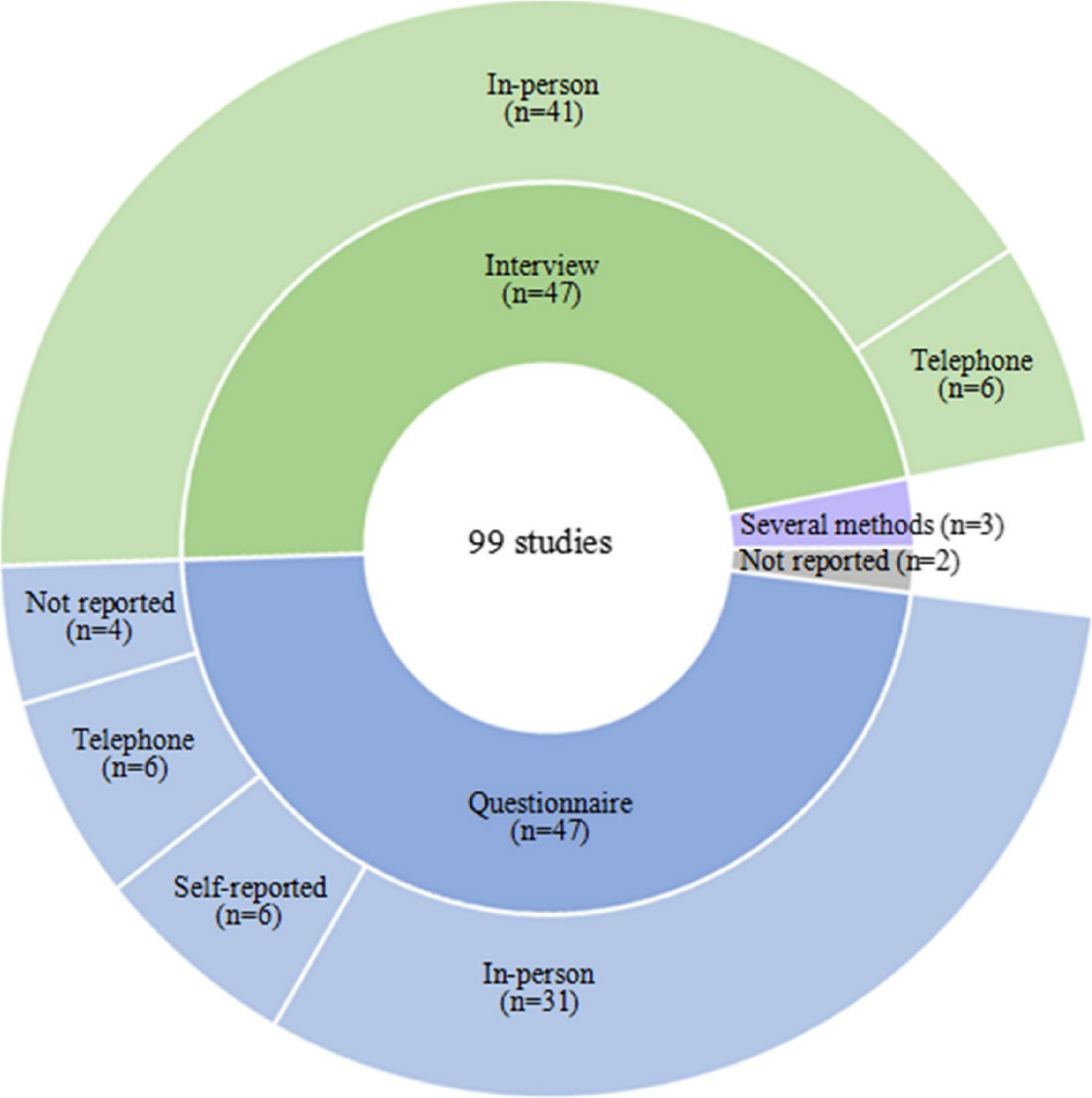
Sunburst chart displaying the distribution of methods used for data collection on patients’ knowledge of the indications for their medications in the included studies

Figure 2 shows detailed information about the methods used to collect data, which most frequently were collected through interviews (*n* = 47) or questionnaires (*n* = 47).

In 12 studies, it was described if patients were allowed to seek assistance from relatives, medication lists or medicine bottles when answering questions. Eight studies assessed participants’ “self-rated” knowledge of indications, i.e., to what extent they felt that they knew the indications for their medications [50, 51, 69, 72, 81, 95, 110, 123], and 28 of the studies described what they compared patients’ responses to in order to assess if patients’ responses on indication were correct, i.e., comparing patient responses to the UpToDate database, general practitioners’ information, medical discharge summaries or the clinical judgment of the reviewer (for details see Supplement 2).

Sixteen studies used previously described methods to assess patients’ knowledge of the indications for their medications, and 83 studies used their own methods or did not cite the methods used. Examples of methods used included the “Medicine Knowledge Assessment Form [46], MedTake [64] and the Care Transitions Measure [81]. The same method was used in a maximum of two of the included studies. In 4 of the 16 studies using methods described in previous publications, the cited publications did not contain information on how to assess knowledge of medication indications [50, 51, 74, 116].

The specific question asked to assess patients’ knowledge of the indications for their medications was reported in 27 of the studies and phrased in 25 different ways (See examples in Table 3). Some questions addressed patients’ self-rated knowledge on a predefined Likert scale, some were open questions, and some were closed questions with categorical response options (for details see Supplement 2).

**Table 3:** Examples of questions asked to assess knowledge of indications, how responses were categorised and reporting of key results

Study ID	Examples of questions	Examples of how responses were categorised	Key results regarding knowledge of indications
Barat 2001	Not reported	*The participants’ answers were evaluated as correct, no knowledge, or wrong*	*Sixty percent (males 64%, females 57%) knew the purpose of treatment for at least 75% of their medications, and 21% (males 24%, females 19%) understood the consequences of omission of a drug or a dose. Only 4% had knowledge of side-effects, and 5% of the toxic risks. No one knew anything about the risk of drug interactions*
Didone 2021	*For what do/will you have to take/use this medication?*	*Answers were compared to the UpToDate database and categorised as correct, incomplete, unknown, or incorrect*	*Sixty-one (52.1%) of the included individuals appropriately recalled 100% of the indications for the medications in use. The appropriate recall of all indications was negatively associated with the number of medications in use* *Of 596 medications identified, 57.0% of the recalled indications were correct, 25.3% were incomplete, 6.7% were unknown, and 10.9% were incorrect. Thus, 82.3% and 17.6% of medication indications were appropriately and inappropriately recalled, respectively. The frequency of inappropriate recall of indications ranged from 9.1% (beta blocking agents) to 33.3% (psychoanaleptics)*
Gama 2021	*Do you know the purpose of this medication? (yes/no). If the patient answered “yes,” then they were asked, What is the purpose?*	*Patients were classified into two groups as follows: lower insight of drug’s purpose (not knowing at least one purpose of their medications) and absent insight of drug’s purpose (not knowing the purposes of any of their medications)*	*Overall, 11.6% of patients did not know the purpose of any of their prescribed drugs, and 40.4% did not know the purpose of at least one prescribed drug* *Of the 1991 prescribed drugs, patients did not know the purpose of 537* *In multivariate analysis, polypharmacy, illiteracy, and cognitive impairment were associated with not knowing the purpose of at least one drug, and illiteracy and insomnia were associated with the misunderstanding of the purpose of all prescribed drugs*
Gwynn 2015	*When I left the hospital, I clearly understood the purpose for taking each of my medicines*	*Strongly disagree, Slightly disagree, Not sure, Slightly agree, and Strongly agree. Their responses were given a numerical value from 1 (strongly disagree) to 5 (strongly agree)*	*I currently understand the purpose of all my medications: Strongly agree 61%, slightly agree 21%, not sure 0%, slightly disagree 15% and strongly disagree 3%*
Fletcher 1979	Not reported	Not reported	*65% of patients (n* = *133) knew the indications for all their medications. Of all medications (n* = *432), 83% of the indications were known* *After assessing a variety of factors that might be associated with knowledge of medications, three emerged that were all inversely related to this variable: age, the number of medical problems, and the number of medications prescribed. These factors were associated with each other, as well as with the three measures of knowledge, so it was impossible truly to separate their effects*
Louis-Simonet 2004	*For what health problem do you take this medication?*	*For each medication, answers were rated as either correct or incorrect. Inability to give an answer was coded as incorrect*	*Knowledge of medication purpose at baseline: 88% in the control service, and 87% in the experimental service. Knowledge of medication purpose at the intervention phase: 89% in the control service, and 96% in the experimental service* *Age (negatively), living alone(negatively) and education(positively) were correlated to knowing purpose of medication*
Son 2016	Not reported	*Patients responded using a 5-point Likert-type scale with scores ranging from 5 to 25*	*Medication knowledge scores M* ± *SD: 16.92* ± *2.51. Medication knowledge was a significant predictor of HRQoL (β* = *.14, p* = *.04, 95% CI* = *[0.01, 0.44])*

Thirty-seven studies reported how they categorised responses. Some studies categorised patients’ knowledge on a Likert scale (between 3 and 6 categories), others reported dichotomised as correct or incorrect, and some reported correct, incorrect or unknown. Sixty-two studies did not report how they categorised participants’ responses. However, based on how the results were reported, we categorised the study participants’ responses as correct or incorrect. Only 17 studies reported both how the questions were phrased and how participants’ responses were categorised.

Details on methods used in all 99 studies can be seen in Supplement 1–2.

### Reporting of results

Knowledge of indications was reported as a proportion of either: 1) all participants (*n* = 65); 2) the total number of medications used by all patients (*n* = 13); or 3) both reporting methods (*n* = 16). Five studies reported a proportion without specifying the denominator.

Eighty-one studies reported the proportion of participants with correct knowledge of the indications for their medications as a fraction of all participants. Of these, 14 reported the proportion of participants who knew all their medications indications, which ranged from 19% (assessed in long-term psychiatric in-patients) to 87% (assessed in adult patients in general practices), with a median of 59% (IQR 51–69%). Similarly, 23 studies reported the proportion of medications where the patients correctly identified the indication in relation to the total number of medications, which ranged from 59 to 94% (median 81%, IQR 70–86%).

The studies assessed many different components of medication knowledge. Of the 95 quantitative studies, 70 studies assessed medication knowledge for several components (median 3, IQR 1–4), such as name (sometimes specified as tradename or generic name) (*n* = 39), side-effects (*n* = 29), dose (*n* = 15), frequency of use (*n* = 13), dosage (*n* = 11), duration of treatment (*n* = 6) and interactions (*n* = 5). In total, 51 combinations of components were identified, with the most common combinations being 'indication’ and ‘side-effects’ (*n* = 10) and ‘indication’ and ‘name’ (*n* = 5).

Eleven studies specified cut-off values for when they considered patients to have good or bad knowledge, e.g. correct knowledge of at least 50% [80] or 75% [17] of their medications. Two studies used a complex scoring system for assessing correct knowledge, where several medication knowledge components (e.g., name, dose, indication) and the number of medications were included in the final score and knowledge assessment [100, 107]. Table 3 shows examples of reporting of study results.

### Knowledge of different drug classes

Out of the 99 included studies, 15 studies reported data on both the overall medication use and the knowledge of different drug classes. Of these 14 studies were observational [5, 6, 27, 41, 48, 55, 56, 60, 73, 77, 90, 91, 104, 124] and one was experimental [101]. How the studies grouped medication classes varied between studies, e.g., one study listed knowledge according to the Anatomical Therapeutic Chemical Classification (ATC) first level categories [90], one study listed knowledge according to ATC second level categories [56] and two studies compared knowledge between different therapeutic categories (not ATC) [27, 91]. Correct knowledge of indication for cardiovascular medications ranged between 62 and 92% in studies [5, 6, 27, 55, 90, 104], and correct knowledge of indication for endocrinological medications ranged between 67 and 97% [5, 6, 27, 55, 91, 104].

### Participant characteristics associated with knowledge of medication indication

Fifty-five studies assessed characteristics associated with better knowledge of medication indications. The most commonly studied characteristics were age (*n* = 36), gender (*n* = 30), educational level (*n* = 29), polypharmacy or number of medications (*n* = 28), marital status (*n* = 7), living situation (*n* = 7), cognitive function (*n* = 7), assistance with medication at home (*n* = 7), comorbidities (*n* = 6), literacy or health literacy (*n* = 6) and income (*n* = 5). Collectively the results were ambiguous as most studies found no association between patient characteristics, however some studies found that high age (in 17 out of 36 studies), male sex (in 6 out of 30 studies), low education (in 10 out of 29 studies) and polypharmacy (in 20 out of 28 studies) were associated with poorer knowledge of medication indications. Whether participants’ knowledge of the indications for their medications is associated with the quality including appropriateness of their treatment was not investigated in any study.

## Discussion

This scoping review synthesised 99 studies on medication knowledge conducted worldwide over the past 45 years, mainly using a cross-sectional design. There was no clear picture from the collective data other than the numerous methods used for collecting, analysing and reporting data also lead to highly heterogenous results.

We found no clear signals for the association between participant characteristics and patients’ knowledge of the indications for their medications, nor did we identify any study investigating whether participants’ knowledge of the indications for their medications was associated with the quality, e.g. appropriateness, of their treatment. Moreover, there seems to be no general consensus across the studies concerning how to assess participants’ knowledge of the indications for their medications. While we expected the use of previously published knowledge assessment tools in different settings and across different patient groups (e.g., patients with low health Literacy, language barriers, elderly patients), we were surprised at how few of these tools were utilised. Nearly all studies developed new methods which was never used in more than two studies. Similarly, there was a lack of consensus regarding whether to report at participant or medication level, and in some studies, it was unclear what the numerator and denominator were. For some of the studies that reported knowledge as a fraction of all patients, it was unclear how they reached the results for patients taking more than one medication (e.g. a study reported that 80% of participants knew the indications for their medications, but it was unclear how this was calculated if patients, for example, knew the indications for three out of five medications).

### Strengths and limitations

To our knowledge, this is the first scoping review of studies assessing patient’ knowledge of the indications for their medications. We conducted a systematic literature search, included publications without language restrictions and contacted authors of conference abstracts to identify all relevant studies. Thus, based on this review it should be possible to improve methods for future research on this important topic.

However, our study also has some limitations. First, the diverse terminology used to convey knowledge about medication indications may have led to the inadvertent omission of relevant studies – particularly if this was not the primary focus of the study. However, when in doubt, we assessed full-text records and included 14 studies where information on medication knowledge was only described in the results section. Second, we excluded 51 studies that only assessed part of participants’ medication use (e.g. studies that only assessed knowledge for random, newly prescribed, or selected types of medications). This means that we may have lost important information, as we had to exclude some qualitative studies, where we might have found a better description of why the patients have more or less knowledge about their medications. However, we excluded these studies because we aimed to assess patients’ knowledge of their total medication use. Last, we originally planned to search reference lists of included studies, Google Scholar, and Web of Science for relevant publications that cited the included studies. However, we did not anticipate including such a high number of studies and decided post hoc that searching these additional sources was not feasible. Despite these limitations, we believe that our scoping review provides a comprehensive and representative map of the field.

### Future research

A single knowledge assessment tool is unlikely to be appropriate for all settings and patient groups, but we believe there is a need to establish a consensus on how best to assess knowledge of indications and report results. For example, by using methodology similar to developing a core outcome set [125, 126]. Further, studies were hampered by inadequate reporting, and we encourage researchers to provide detailed descriptions of their methods in future publications. This should include the phrasing of questions for assessing knowledge, the criteria for determining the correctness of the knowledge, and the categorisation of patients’ responses. We also recommend that future studies report knowledge of indications both at the level of study participants and total medication use.

## Conclusion

We did not identify any established scientific standard for assessing patients’ knowledge of the indications for their medications. The wide range of study methodologies and reporting styles observed call for a methodological consensus in this research field. Estimates of correct knowledge varied widely between studies, but whether this was due to differences in study populations or study methodology could not be determined. Furthermore, we did not identify any study investigating whether participants’ knowledge of the indications for their medications was associated with the quality, e.g. appropriateness, of their treatment.

## Supplementary Information

Supplementary Material 1.

Supplementary Material 2.

## Data Availability

All data generated or analysed during this study are included in this published article [and its supplementary information files].

## Data availability

Data is provided within the manuscript or supplementary information files.
