# Supplementary material for: Patients’ knowledge of the indications for their medications – a scoping review
Source: BMC Health Serv Res. 2024 Oct 8;24:1195. doi: 10.1186/s12913-024-11685-7 (PMC11460199; doi:10.1186/s12913-024-11685-7)
Supplement: Supplementary file 1 — Supplementary Material 1. [file 12913_2024_11685_MOESM1_ESM.docx]

**Appendix 1**

**Search strategy**

Search strategy for Medline, Ovid: ((elderly or older or patient* or participant*) adj8 (know* or understand*) adj5 (drug* or medicine* or medication*) AND (purpose* or indication* or “reason* for use” or “reason* for taking”)).mp. or exp Patient Medication Knowledge/

Search strategy for EMBASE, Ovid: ((elderly or older or patient* or participant*) adj8 (know* or understand*) adj5 (drug* or medicine* or medication*)).mp. [mp=title, abstract, heading word, drug trade name, original title, device manufacturer, drug manufacturer, device trade name, keyword heading word, floating subheading word, candidate term word]

Search strategy for CINAHL (the Cumulative Index to Nursing and Allied Health Literature) via Ebsco: ((elderly or older or patient* or participant*) N8 (know* or understand*) N5 (drug* or medicine* or medication*)) OBS secect all text

Search strategy for Psychinfo via Ebsco: ((elderly or older or patient* or participant*) N8 (know* or understand*) N5 (drug* or medicine* or medication*)) OBS secect all text OR patient medication knowledge OBS select MA MeSH Subject Heading

Search strategy for Cochrane Library: (MeSH descriptor (patient Medication Knowledge) explode all trees) OR (((elderly or older patient* or participant*) NEAR/8 (know* or understand*) NEAR/5 (drug* or medicine* or medication*)) AND (purpose* or indication* or "reason for use” or “reason for taking”))

**Reporting of methods used to assess patients' knowledge of the indications for their medications based on study aims**

| **Method Included description of** | **Primary aim**  (n=59) | **Not primary aim**  (n=27) | **Not part of the aim**  (n=13) |
| --- | --- | --- | --- |
| The phrasing of the question asked to access knowledge of indication | 29% | 22% | 31% |
| If the patient had access to help when answering | 15% | 4% | 15% |
| If patients’ answers were compared to something to assess the correctness | 29% | 37% | 8% |
| How answers were categorized/options for answering | 41% | 37% | 23% |
| How knowledge was calculated per patient when patients use more than one drug | 36% | 30% | 8% |
| When knowledge was assessed as adequate | 15% | 7% | - |
| Total | 27% (97/354) | 23% (37/162) | 13% (10/78) |
